# Treatments used by chronic obstructive pulmonary disease patients in Brazil: National Survey of Health, 2013

**DOI:** 10.11606/s1518-8787.2022056004090

**Published:** 2022-11-18

**Authors:** Gabriela Ávila Marques, Paula Duarte de Oliveira, Marina Montzel, Ana Maria Baptista Menezes, Deborah Carvalho Malta, Luciana Monteiro Vasconcelos Sardinha, Fernando César Wehrmeister

**Affiliations:** I Universidade Federal de Pelotas Faculdade de Medicina Programa de Pós-Graduação em Epidemiologia Pelotas RS Brasil Universidade Federal de Pelotas. Faculdade de Medicina. Programa de Pós-Graduação em Epidemiologia. Pelotas, RS, Brasil; II Universidade Federal de Pelotas Faculdade de Medicina Departamento de Medicina Social Pelotas RS Brasil Universidade Federal de Pelotas. Faculdade de Medicina. Departamento de Medicina Social. Pelotas, RS, Brasil; III Universidade Federal de Minas Gerais Escola de Enfermagem Departamento de Enfermagem Materno-Infantil e Saúde Pública Belo Horizonte MG Brasil Universidade Federal de Minas Gerais. Escola de Enfermagem. Departamento de Enfermagem Materno-Infantil e Saúde Pública. Belo Horizonte, MG, Brasil; IV Ministério da Saúde Brasília DF Brasil Ministério da Saúde. Brasília, DF, Brasil

**Keywords:** Pulmonary Disease, Chronic Obstructive, epidemiology, Bronchitis, Chronic, Pulmonary Emphysema, Disease Management, Health Surveys

## Abstract

**OBJECTIVE:**

To estimate the prevalence of treatments used for the management of chronic obstructive pulmonary disease (COPD) in the Brazilian adult population.

**METHODS:**

A population-based cross-sectional study with data from the 2013 Brazilian National Survey of Health, including individuals aged 40 years or older, with a self-reported medical diagnosis of COPD, chronic bronchitis and/or emphysema, who were asked about treatments used for disease management.

**RESULTS:**

A total of 60,202 adults were interviewed, of which 636 were 40 years of age or older and had reported a medical diagnosis of COPD, emphysema, or chronic bronchitis. Less than half (49.4%) of the diagnosed population reported using some type of treatment, with differences regarding the macro-region of the country (South 53.8% – Northeast 41.2%, p = 0.007). Pharmacological treatment was the most reported, and emphysema patients had the highest proportion of those undergoing more than one type of treatment. Among the individuals who reported having only chronic bronchitis, 55.1% (95%CI: 48.7–61.4) used medication, 4.7% (95%CI: 2.6–8.3) underwent physical therapy, and 6.0% (95%CI: 3.6–9.9) oxygen therapy. On the other hand, among the emphysema patients, 44.1% (95%CI: 36.8–51.7) underwent drug treatment, 8.8% (95%CI: 5.4–14.2) physical therapy, and 10.0% (95%CI: 6.3–15.6) oxygen therapy.

**CONCLUSION:**

The prevalence of treatments for COPD management was below ideal in 2013. The pharmacological treatment was the main type of treatment, followed by oxygen therapy and physical therapy.

## INTRODUCTION

Chronic non-communicable diseases, including chronic respiratory diseases, are the main causes of morbidity and mortality in Brazil. Chronic obstructive pulmonary disease (COPD) is the third leading cause of death in the world, accounting for more than three million deaths per year^1,2^.

Encompassing chronic bronchitis and pulmonary emphysema, COPD is characterized by chronic obstruction of the lower airways, causing symptoms such as wheezing, dyspnea, and productive cough^2^. Minimizing exposure to risk factors, in association with patient health education and adherence to the recommended treatment, is essential in reducing the frequency and severity of exarcebations^2^.

The COPD treatment protocol in Brazil is followed by the entire network of the Unified Health System (SUS). Among the different types of treatment, the pharmacological is considered one of the main pillars within the management of the disease^3^. Respiratory physical therapy, oxygen therapy, and/or the use of noninvasive ventilation, according to severity and indication, are also strategies with great potential to minimize respiratory symptoms, improve quality of life, and reduce the risk of mortality^4^.

Ignoring or undergoing an inadequate treatment can lead to unnecessary hospitalizations, as well as higher direct and indirect costs to the population and high mortality^5^. Only a small portion of the population undergoes some type of treatment, according to a survey^6^ on COPD, which concluded that, in Brazil, especially in São Paulo, 83.3% of people with chronic obstructive pulmonary disease did not receive pharmacological treatment.

In this scenario, the aim of this study is to estimate the prevalence of treatments used for the management of COPD according to sociodemographic, behavioral, and health services variables, in a representative sample of the adult population of Brazil.

## METHODS

### Study Design and Data Source

This cross-sectional, population-based study used data from the Brazilian 2013 National Survey of Health (*Pesquisa Nacional de* Saúde – PNS). The PNS is carried out by the Brazilian Institute of Geography and Statistics (IBGE), in partnership with the Ministry of Health, evaluating the performance of the system and the health conditions of Brazilians. To ensure the representativeness of the national population, cluster sampling was used in three stages: census tracts, households, and adult individuals (18 years or older)^7^.

The questionnaire, conducted by the Family Health team and health agents during home visits, included questions about the household and the characteristics of all its residents, as well as individual health related questions. For this study, the module entitled “Chronic Diseases” was used, in which the population was questioned about the medical diagnosis of COPD and the treatments used to control the disease. More PNS details are available in previous publications^8^.

For this study, we included individuals aged 40 years or older (following the age cutoff point of other COPD studies)^9^, who responded positively to question Q116: “Has a doctor ever given you a diagnosis of lung disease, such as pulmonary emphysema? (no/yes), chronic bronchitis? (no/yes), or COPD (chronic obstructive pulmonary disease)? (no/yes).” Since was possible to differentiate the presence of chronic bronchitis and emphysema, a dichotomous variable was created for each of these diseases.

### Treatments Used by Patients with the Disease

Individuals who reported having COPD were asked about treatments in question Q118: “What do you currently do for the lung disease?” Among the alternatives available for the answer were: “a. Uses medications (inhalers, aerosol, or pills); b. Uses oxygen; c. Respiratory physical therapy; d. Other,” and the interviewee can answer more than one option. Dichotomous variables (yes/no) were created for each of the listed treatments and a variable for those who perform any of the three types of treatment.

### Exposure Variables

The treatments were described according to the following variables: gender (man/woman), age in years (40-49, 50-59, 60 or more), race/skin color (white, black, mixed, yellow, indigenous), smoker (no/yes), level of education (illiterate, incomplete Primary Education, complete Primary Education or incomplete Secondary Education, complete Secondary or complete Higher Education, complete Higher education or more), place of residence (urban/rural), macro-region of the country (North, Northeast, Southeast, South, Midwest), wealth index, and variables related to access and use of health services.

The analysis of the main components was used for the construction of the wealth index^10^, regardless of the place of residence. Among the available variables, 14 that referred to the presence or absence of a certain items and commodities were selected, namely: number of cars, DVD player, washing machine, microwave, telephone, computer, mobile phone, improved source of water supply, electricity source, internet access, number of bathrooms and use of appropriate materials in the construction of the house (roof, wall, floor). Subsequently, the wealth index was divided into quintiles, with the first quintile (Q1) comprised the poorest individuals and the highest quintile (Q5), the richest.

The variables for access and use of health services were registration of the household in the Family Health Unit (FHU) (no/yes) and possession of health insurance (no/yes).

### Statistical Analysis

The sample was described by means of absolute and relative frequencies, according to exposure variables. When considering the type of treatment, stratified by the type of disease (COPD, chronic bronchitis, or emphysema), the estimates were expressed in relative frequency, with intervals of 95% confidence (95%CI), considering the sample design of the research, by means of the *svy* command. The comparison of the prevalence of each treatment according to the exposure variables was performed using the χ^2^ test, results with a p-value < 0.05 were considered as statistically significant.

To visualize the simultaneous use of more than one type of treatment, Venn diagrams were constructed for each subgroup of disease. In which, each of the circumferences represents one of the treatments (pharmacological, oxygen therapy, or physiotherapy). Thus, we can view the proportion of each intersection.

All data were analyzed using the Stata program, version 15.0 (StataCorp LP, College Station, Texas, United States).

### Ethical Aspects

The PNS was approved by the National Research Ethics Commission. The participation was voluntary, and the confidentiality of the obtained information was guaranteed. The participants who agreed to participate signed the informed consent form in two copies. The data are publicly available on the website of the Brazilian Institute of Statistical Geography (IBGE)^8^.

## RESULTS

Among the 60,202 adults interviewed, 31,612 (35.2%) were 40 years of age or older, of which 636 (2.0%) reported a medical diagnosis of COPD. The population prevalence of chronic bronchitis in individuals aged 40 years or more was 0.9%, and the prevalence of emphysema was 0.7%, isolated. Table 1 describes the sample according to the self-reported diagnosis and the undergoing treatment. Among all types of diseases evaluated, most individuals were 60 years or older, white, nonsmoker, had low educational levels, were resident in the urban area, with a household registered in the FHU, without health insurance, and considered their health to be very poor. Regarding gender, patients with COPD and chronic bronchitis were mostly women, whereas among those with emphysema were mostly men (Table 1). Regarding the use of any treatment, 47% reported some treatment for COPD, 56% for chronic bronchitis, and 48% for emphysema. In patients with COPD, there were differences regarding the macro-region of the country (South 53.8%, Northeast 41.2%) and self-assessment of health conditions (poor 67.1% and very good 23.1%). Among patients with chronic bronchitis, a similar pattern was found for the macro-region of the country, and a higher proportion of those undergoing any treatment were among those with a household not registered in the FHS (66.3%). Among emphysema patients, men underwent more treatment than women (53.1% versus 45.9%).

**Table 1:** Description of the sample of Brazilians with self-reported medical diagnosis for chronic obstructive pulmonary disease (COPD), chronic bronchitis, or emphysema, and who reported undergoing some type of treatment, according to sociodemographic, behavioral, and health services variables. PNS, 2013.

Characteristics	COPD (n = 636)			Chronic bronchitis (n = 234)			Emphysema (n = 170)		
		
	n	%	Performs some treatment (%)	n	%	Performs some treatment (%)	n	%	Performs some treatment (%)
Gender			p = 0.502			p = 0.967			**p = 0.017**
Man	269	42.3	47.2	78	33.3	48.7	96	56.5	53.1
Woman	367	57.7	51.0	156	66.7	62.8	74	43.5	45.9
Age, years			p = 0.458			p = 0.905			p=0.625
40–49	133	20.9	46.6	59	25.2	55.9	16	9.4	37.5
50–59	151	23.7	49.0	63	26.9	60.3	35	20.6	42.9
≥ 60	352	55.4	50.6	112	47.9	58.0	119	70.0	53.8
Race/Skin color			p = 0.105			p = 0.422			p = 0.203
White	332	52.2	51.8	120	51.3	61.7	92	54.1	53.3
Black	50	7.9	42.0	21	9.0	52.4	5	2.9	40.0
Asian	5	0.8	40.0	1	0.4	100.0	1	0.6	0.0
Mixed	242	38.1	47.5	91	38.9	54.9	71	41.8	47.9
Indigenous	7	1.1	57.1	1	0.4	0.0	1	0.6	0.0
Currently smoking			p = 0.104			p = 0.357			p = 0.323
No	482	75.8	52.3	172	73.5	64.5	127	74.7	55.1
Yes	154	24.2	40.3	62	26.5	40.3	43	25.3	34.9
Educational level			p = 0.081			p = 0.113			p = 0.055
Illiterate	144	22.6	52.1	54	23.1	55.6	43	25.3	58.1
Incomplete primary education	245	38.5	45.7	91	38.9	48.4	66	38.8	43.9
Complete primary education and incomplete secondary education	70	11.0	48.6	22	9.4	72.7	20	11.8	55.0
Complete secondary education or incomplete higher education	102	16.0	52.9	36	15.4	75.0	27	15.9	55.6
Complete higher education	75	11.8	52.0	31	13.3	61.3	14	8.2	35.7
Place of residence			p = 0.300			p = 0.639			p = 0.195
Urban	550	86.5	49.8	198	84.6	59.1	150	88.2	50.0
Rural	86	13.5	46.5	36	15.4	52.8	20	11.8	50.0
Macro-region			**p = 0.007**			**p < 0.001**			p = 0.094
North	80	12.6	50.0	29	12.4	51.7	20	11.8	60.0
Northeast	131	20.6	41.2	33	14.1	45.5	39	22.9	38.5
Southeast	193	30.4	51.8	75	32.1	70.1	44	25.9	52.3
South	143	22.5	53.8	62	26.5	56.5	41	24.1	53.7
Midwest	89	14.0	48.3	35	15.0	51.4	26	15.3	50.0
Wealth quintile			p = 0.185			p = 0.429			p = 0.071
Q1 (most poor)	94	14.8	40.4	33	14.1	42.4	24	14.1	37.5
Q2	159	25.0	49.7	58	24.8	51.7	49	28.8	51.0
Q3	153	24.1	48.4	62	26.5	59.7	42	24.7	50.0
Q4	102	16.0	48.0	38	16.2	68.4	18	10.6	44.4
Q5 (most riche)	128	20.1	57.8	43	18.4	67.4	37	21.8	59.5
Household registered with FHU			p = 0.625			**p < 0.001**			p = 0.254
No	277	43.6	47.3	98	41.9	66.3	73	42.9	46.6
Yes	359	56.5	51.0	136	58.1	52.2	97	57.1	52.6
Health insurance			p = 0.635			p = 0. 296			p = 0.499
No	407	64.0	50.4	153	65.4	57.5	110	64.7	51.8
Yes	229	36.0	47.6	81	34.6	59.3	60	35.3	46.7
Self-assessment of health			**p = 0.023**			p = 0.461			p = 0.283
Very good	26	4.1	23.1	9	3.9	22.2	4	2.4	0
Good	181	28.5	40.9	58	24.8	53.4	42	24.7	47.6
Regular	300	47.2	50.7	119	50.9	58.8	83	48.8	48.2
Bad	85	13.4	67.1	33	14.1	72.7	28	16.5	64.3
Very bad	44	6.9	56.8	15	6.4	60.0	28	7.7	53.8

FHU: family health unit.

The Figure shows the proportions of each treatment (pharmacological, physical therapy, and oxygen therapy) in the three subgroups. In all, the pharmacological treatment was the most mentioned. Patients with emphysema had the highest proportion of those undergoing more than one type of treatment. Among the individuals evaluated, 44.8% reported using medications for the management of the disease, with differences only by macro-region of the country, with the Northeast with the lowest prevalence (27.5%; 95%CI: 18.3–39.1) and the Southeast with the highest (55.2%; 95%CI: 44.8–65.1) (Table 2).

**Figure:**
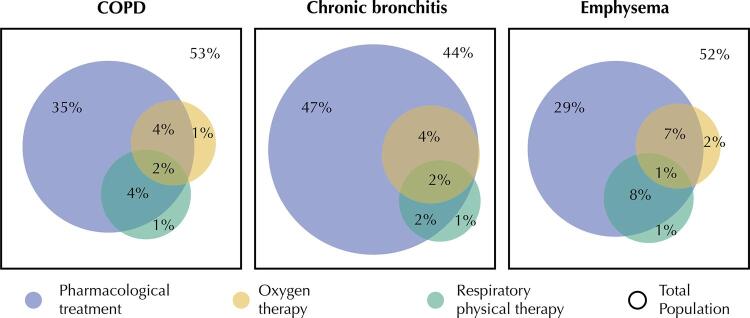
Venn diagram with the appropriate proportions of treatments used for the control of chronic obstructive pulmonary disease (COPD). PNS, 2013.

**Table 2:** Distribution of the prevalencea of medication use in Brazilians with chronic obstructive pulmonary disease (COPD), chronic bronchitis, or emphysema, according to sociodemographic, behavioral, and health services variables. PNS, 2013.

Characteristics	Medication					

	COPD		Chronic bronchitis		Emphysema	
		
	%	95%CI	%	95%CI	%	95%CI
Gender		p = 0.625		p = 0.979		**p = 0.039**
Man	49.5	39.6–59.4	62.7	47.4–75.8	53.7	38.0–68.6
Woman	46.1	37.6–54.9	62.4	49.4–73.9	29.1	15.9–47.1
Age, years		p = 0.357		p = 0.941		p = 0.307
40–49	47.2	33.8–61.0	64.7	41.8–82.4	40.7	14.7–73.3
50–59	39.1	28.0–51.4	60.0	41.6–75.9	24.1	11.0–44.9
≥ 60	50.8	42.0–59.6	62.4	48.4–74.7	48.0	33.6–62.8
Race/Skin color		**p = 0.039**		p = 0.363		**p = 0.015**
White	54.5	45.9–62.8	67.9	55.4–78.2	52.3	36.5–67.6
Black	30.6	14.5–53.4	57.4	27.6–82.6	6.2	0.6–41.4
Asian	66.4	13.2–96.2	100.0	–	0.0	–
Mixed	38.0	28.0–49.0	53.3	35.3–70.4	30.8	18.1–47.4
Indigenous	45.4	7.1–90.0	0.0	–	0.0	–
Currently smoking		p = 0.134		p = 0.366		p = 0.413
No	50.6	42.9–58.2	65.3	53.0–75.7	46.9	33.2–61.2
Yes	38.4	26.3–52.2	54.8	35.3–72.8	34.8	15.4–61.0
Educational level		p = 0.106		p = 0.125		p = 0.168
Illiterate	51.6	38.0–65.0	50.1	29.3–70.8	66.1	42.4–83.7
Incomplete primary education	42.8	32.8–53.4	54.2	38.9–68.8	36.8	21.5–55.3
Complete primary education and incomplete secondary education	37.2	20.3–57.9	68.9	33.2–90.8	30.3	9.4–64.6
Complete secondary education or incomplete higher education	47.2	31.9–63.0	73.3	49.5–88.5	39.1	16.3–67.9
Complete higher education	69.4	52.0–82.6	82.3	63.0–92.7	43.6	16.1–75.7
Place of residence		p = 0.254		p = 0.69		**p = 0.009**
Urban	48.4	41.5–55.4	62.9	52.1–72.6	45.4	33.1–58.3
Rural	38.6	25.0–54.2	58.2	36.8–76.9	14.9	5.2–36.0
Macro-region		**p = 0.005**		**p < 0.001**		p = 0.087
North	33.0	19.7–49.7	27.3	12.0–50.9	60.0	31.4–83.1
Northeast	27.5	18.3–39.1	32.6	14.8–57.4	17.4	7.2–36.3
Southeast	55.2	44.8–65.1	79.8	67.5–88.3	42.8	23.8–64.1
South	49.3	37.2–61.4	49.8	32.3–67.3	57.8	36.4–76.7
Midwest	44.0	31.2–57.6	55.5	35.5–73.9	44.7	23.1–68.5
Wealth quintile		p = 0.138		p = 0.444		**p = 0.044**
Q1 (most poor)	32.9	20.7–48.1	42.4	20.8–67.3	21.3	6.7–50.5
Q2	42.2	29.8–55.6	59.9	39.9–77.1	39.6	20.0–63.1
Q3	47.5	34.7–60.6	56.9	36.7–74.9	44.8	21.8–70.3
Q4	44.0	28.6–60.7	65.6	41.3–83.8	24.3	7.3–56.7
Q5 (most riche)	61.1	48.5–72.4	74.1	52.9–87.9	76.9	57.9–88.9
Household registered with FHU		p = 0.473		**p < 0.001**		p = 0.455
No	50.3	40.8–59.7	79.3	67.7–87.5	38.4	22.3–57.5
Yes	45.7	37.2–54.4	49.5	36.9–62.2	47.7	32.3–63.6
Health insurance		p = 0.505		p = 0.278		p = 0.738
No	49.3	40.9–57.7	66.9	53.9–77.7	42.5	28.7–57.7
Yes	44.7	34.8–55.1	55.8	39.7–70.7	47.0	26.9–68.2
Self-assessment of health		p = 0.051		p = 0.493		p = 0.299
Very good	62.6	29.9–86.8	77.9	32.6–96.2	0.0	–
Good	34.8	24.4–47.0	58.6	38.6–76.2	32.4	13.9–58.7
Regular	48.2	39.1–57.5	57.4	42.9–70.8	45.0	29.2–61.8
Bad	58.4	41.9–73.3	77.5	55.9–90.3	42.5	18.6–70.4
Very bad	67.4	45.1–83.8	71.6	37.2–91.5	73.9	35.5–93.6

FHU: family health unit.

^a^ Estimated by the weight of the individual selected for the sample.

Values with statistical significance are shown in bold.

The physical therapy treatment (Table 3) was performed by 6.9% (95%CI: 5.2–9.2), 4.7% (95%CI: 2.6–8.3), and 8.8% (95%CI: 5.4–14.2) of patients with COPD, chronic bronchitis, and among emphysema, respectively. Only 1.3% (95%CI: 0.5–3.0) of current smokers underwent physical therapy compared to 6.8% of nonsmokers (p < 0.001). Among the emphysema patients, differences per quintile of richness were observed; the richest quintile had 23.7% (95%CI: 9.3–48.7), while in the other quintiles this prevalence was lower than 6% (p = 0.003) (Table 3).

**Table 3:** Distribution of prevalencea regarding physical therapy treatment in Brazilians with chronic obstructive pulmonary disease (COPD), chronic bronchitis or emphysema, according to sociodemographic, behavioral, and health services variables. PNS, 2013.

Characteristics	Physical therapy					

	COPD		Chronic bronchitis		Emphysema	
		
	%	95%CI	%	95%CI	%	95%CI
Gender		p = 0.328		p = 0.569		p = 0.884
Man	6.9	3.6–12.8	5.7	1.1–25.8	6.3	2.5–15.4
Woman	4.4	2.4–8.1	3.3	1.3–8.2	7.1	1.8–24.1
Age (years)		p = 0.886		**p = 0.012**		p = 0.09
40–49	4.9	1.6–13.7	2.0	0.6–6.7	14.2	2.0–57.7
50–59	4.7	1.6–12.9	0.1	0.0–1.0	18.9	5.3–49.4
≥ 60	6.0	3.4–10.4	6.6	2.4–17.2	3.2	1.4–7.2
Race/Skin color		**p = 0.010**		p = 0.244		p = 0.14
White	4.5	2.3–8.6	4.5	1.3–14.2	4.7	1.3–14.8
Black	22.7	8.2–49.1	19.6	2.9–66.2	31.2	3.6–84.5
Asian	0.0	–	0.0	–	0.0	–
Mixed	4.2	2.3–7.7	1.8	0.6–6.0	7.4	2.7–18.7
Indigenous	0.0	–	0.0	–	0.0	–
Currently smoking		**p < 0.001**		**p = 0.025**		p = 0.092
No	6.8	4.3–10.8	5.3	2.1–12.9	8.0	3.4–17.7
Yes	1.3	0.5–3.0	0.7	0.1–4.7	2.4	0.7–8.1
Educational level		p = 0.690		p = 0.476		p = 0.841
Illiterate	4.7	1.6–13.1	2.8	0.4–18.0	7.1	1.3–31.5
Incomplete primary education	6.3	3.1–12.6	6.0	1.4–21.7	5.8	1.5–19.4
Complete primary education and incomplete secondary education	2.4	0.7–7.6	11.4	0.1–8.8	4.3	0.7–22.7
Complete secondary education or incomplete higher education	4.4	1.7–10.7	0.2	0.0–1.5	7.6	1.8–26.6
Complete higher education	7.8	3.1–18.2	6.1	1.4–22.5	17.0	36.3–52.5
Place of residence		p = 0.818		p = 0.238		p = 0.525
Urban	5.4	3.4–8.7	3.5	1.2–9.8	7.0	3.1–14.6
Rural	6.3	1.9–19.2	10.0	2.1–36.1	0.0	–
Macro-region		p = 0.508		p = 0.246		p = 0.957
North	10.5	4.1–24.4	14.7	4.0–41.9	9.4	1.8–37.8
Northeast	3.3	1.2–8.6	0.0	–	5.8	13.0–22.1
Southeast	4.6	2.2–9.4	2.5	0.7–8.9	5.7	1.5–19.6
South	7.6	3.2–17.0	8.5	2.2–27.7	7.2	1.4–29.5
Midwest	6.6	2.1–18.5	0.0	–	10.2	1.9–39.3
Wealth quintile		p = 0.480		p = 0.222		**p = 0.003**
Q1 (most poor)	1.8	3.8–8.0	0.0	–	1.5	0.2–11.2
Q2	4.0	1.7–8.8	4.1	0.8–18.0	0.2	0.0–1.2
Q3	4.4	1.6–11.2	4.4	1.2–14.8	5.5	0.8–29.3
Q4	8.5	3.1–21.1	11.7	2.3–43.5	3.1	0.8–11.2
Q5 (most riche)	7.0	3.1–15.2	0.5	0.1–3.7	23.7	9.3–48.7
Household registered with FHU		p = 0.795		p = 0.762		p = 0.301
No	5.1	2.6–9.8	4.7	1.7–12.6	4.0	1.5–10.8
Yes	5.8	3.1–10.4	3.6	0.8–14.7	8.3	3.1–20.5
Health insurance		p = 0.528		p = 0.672		p = 0.322
No	4.9	2.5–9.4	4.6	1.4–14.1	4.8	1.4–14.9
Yes	6.5	3.7–11.3	3.2	0.9–10.9	10.3	3.6–26.3
Self-assessment of health		p = 0.772		p = 0.477		p = 0.79
Very good	0.0	–	0.0	–	0.0	–
Good	4.7	1.6–12.9	9.2	2.1–31.9	4.8	1.5–14.3
Regular	5.7	3.0–10.4	2.3	0.7–7.6	7.2	2.5–19.1
Bad	8.6	3.2–21.2	3.2	0.8–12.5	10.9	1.5–49.3
Very bad	5.1	1.6–15.4	4.0	0.5–26.3	2.1	0.2–15.6

FHU: family health unit.

^a^ Estimated by the weight of the individual selected for the sample.

Values with statistical significance are shown in bold.

Oxygen treatment was used by 7.2% (95%CI: 5.5–9.5) of COPD patients, 6.0% (95%CI 3.6–9.9) of individuals with chronic bronchitis, and 10.0% (95%CI: 6.3–15.6) with emphysema. Table 4 shows the distribution of the prevalence of oxygen therapy. Furthermore, oxygen use was higher among illiterate patients who reported COPD (14.4%).

**Table 4:** Distribution of the prevalencea for oxygen therapy in Brazilians with chronic obstructive pulmonary disease (COPD), chronic bronchitis, or emphysema, according to sociodemographic, behavioral, and health service variables. PNS, 2013.

Characteristics	Oxygen inhalation therapy					

	COPD		Chronic bronchitis		Emphysema	
		
	%	95%CI	%	95%CI	%	95%CI
Gender		**p = 0.050**		p = 0.626		**p < 0.001**
Man	10.5	5.4–19.5	8.3	2.4–25.4	17.0	7.2–35.3
Woman	4.1	1.9–8.4	5.6	1.9–15.4	2.2	0.8–5.6
Age, years		p = 0.309		p = 0.053		p = 0.896
40–49	4.2	1.3–12.2	1.3	0.3–5.8	13.6	2.3–51.5
50–59	4.6	1.6–12.7	4.1	1.1–14.1	14.7	3.2–47.5
≥ 60	8.8	4.7–15.9	9.9	3.7–23.6	10.2	3.4–26.6
Race/Skin color		p = 0.480		p = 0.784		p = 0.163
White	8.8	4.7–15.9	7.7	2.6–20.6	13.3	5.1–30.8
Black	8.3	1.5–34.4	1.6	0.2–11.5	31.2	3.6–84.5
Asian	0.0	–	0.0	–	0.0	–
Mixed	3.5	1.8–6.8	5.1	1.9–12.9	2.7	1.1–6.3
Indigenous	0.0	–	0.0	–	0.0	–
Currently smoking		**p < 0.001**		**p < 0.001**		**p < 0.001**
No	8.9	5.3–14.6	8.5	3.7–18.4	14.5	6.4–29.7
Yes	0.7	0.3–1.9	0.5	0.1–3.6	0.8	0.1–4.0
Educational level		**p = 0.012**		p = 0.275		p = 0.332
Illiterate	14.4	6.4–29.2	11.7	2.7–38.8	21.3	6.4–51.9
Incomplete primary education	7.7	3.6–15.4	9.3	3.4–23.2	10.9	3.3–30.3
Complete primary education and incomplete secondary education	0.7	0.1–3.3	2.0	0.4–10.5	0.0	–
Complete secondary education or incomplete higher education	1.7	0.5–5.1	0.0	–	24.5	0.5–11.0
Complete higher education	2.1	0.6–6.7	1.4	0.2–9.9	0.0	–
Place of residence		p = 0.546		p = 0.772		p = 0.625
Urban	7.1	4.1–11.9	6.6	2.7–14.9	11.0	4.6–24.1
Rural	5.3	2.4–11.3	5.2	1.3–18.9	15.3	5.1–38.0
Macro-region		p = 0.280		p = 0.756		p = 0.075
North	6.4	2.1–17.8	8.0	1.2–39.2	13.6	3.1–43.4
Northeast	2.5	1.1–5.7	2.1	0.4–11.3	1.7	0.4–6.9
Southeast	9.2	4.5–18.0	8.0	2.7–21.1	19.7	6.9–44.7
South	5.3	1.8–14.9	5.3	0.7–29.7	7.4	1.8–25.6
Midwest	8.1	3.8–16.4	6.8	1.5–25.1	1.8	0.2–12.2
Wealth quintile		p = 0.366		**p = 0.017**		p = 0.343
Q1 (most poor)	3.9	1.4–10.5	0.6	0.1–4.3	8.2	1.8–30.7
Q2	3.1	1.3–7.1	1.1	0.2–7.5	1.4	0.4–4.9
Q3	11	4.6–24.3	10.6	2.9–32.2	17.7	4.8–48.1
Q4	8.5	3.2–20.7	18.4	5.8–45.2	10.2	1.5–46.1
Q5 (most riche)	6.0	1.7–19.0	0.9	0.1–6.6	18.7	3.9–56.6
Household registered with FHU		p = 0.524		p = 0.571		p = 0.648
No	5.7	2.4–12.6	5.0	2.0–12.0	8.5	1.5–35.6
Yes	7.9	14.5–	7.5	2.4–21.3	13.0	5.1–29.1
Health insurance		p = 0.646		**p = 0.002**		p = 0.558
No	7.6	4.1–13.6	9.8	4.1–21.7	9.3	3.2–24.5
Yes	5.9	2.3–14.1	1.2	0.3–4.9	14.9	4.2–41.5
Self-assessment of health		p = 0.295		**p = 0.040**		p = 0.085
Very good	0.0	–	0.0	–	0.0	–
Good	4.1	1.3–12.3	7.0	1.2–31.1	1.3	0.3–6.7
Regular	6.4	2.9–13.6	4.5	1.9–10.4	12.5	3.8–33.7
Bad	12.3	4.9–27.6	1.8	0.2–12.7	28.1	9.3–59.8
Very bad	18.0	4.6–50.4	39.6	8.8–81.6	3.0	0.6–13.9

FHU: family health unit.

^a^ Estimated by the weight of the individual selected for the sample.

Values with statistical significance are shown in bold.

## DISCUSSION

The proportion of Brazilians with chronic obstructive pulmonary disease who declared to undergo some treatment was below ideal, in 2013, in the three subgroups analyzed (COPD, chronic bronchitis, and emphysema). We found a predominance of women among patients with COPD and chronic bronchitis, differently from other studies, possibly as a result of erroneous interpretations related to other respiratory diseases, whether chronic (as in the case of asthma), or acute (acute bronchitis). Historically, women use more health services when compared with men, with a higher chance of obtaining a diagnosis^11^.

Pharmacological treatment was the most prevalent (44.7%), followed by oxygen therapy (7.2%) and physical therapy (6.9%). Additionally, among those who used at least one of the types of treatment questioned, important differences were observed in gender, race/skin color, history of smoking, educational level, macro-region of the country, as well as wealth index and self-assessment of health condition. In general, while the Southeast region had the highest prevalence of different types of treatment, the Northeast region had the lowest/worst.

Noncommunicable chronic diseases correspond to 72% of the causes of death in Brazil, 5.8% of which occur due to chronic respiratory diseases^12^. Many of these deaths could have been avoided with greater attention and investment in the diagnostic-indication-adherence triad of the therapeutic system. Lower adherence to the recommended treatment, especially pharmacological, is associated with an increase in up to 58% in the risk of hospitalization and up to 40% in death^13^. Additionally, continuous use of drugs is associated with a survival of up to five years in patients with moderate or severe COPD^14^.

Although many advances have occurred in recent years, smoking cessation is still the main factor to treat and control the disease^15,16^. In our study, despite the high prevalence of smokers, they were the ones who underwent the least amount of treatments, with well-marked differences.

Chronic bronchitis and emphysema usually occur simultaneously, but have different definitions and the same individual may present different degrees of impairment^17^. In this analysis, only a small portion of the population performed combined therapies (Figure), especially the one resulting from the articulation between pharmacological and physical therapy, which, unlike oxygen therapy, is more frequent in less advanced stages of the disease^3^.

A study conducted with data from the *Pesquisa Nacional sobre Acesso, Utilização e Promoção do uso Racional de Medicamentos no Brasil* (PNAUM – National Survey on Access, Use, and Promotion of Rational Use of Medicines in Brazil)^18^ observed that, among individuals diagnosed with chronic respiratory diseases, only 41.9% reported having indication for pharmacological treatment. In these cases, only 77.1% used the medications, suggesting a significant gap between indication and treatment adherence, influencing the control of morbidity and mortality of the disease^18,19^. Similarly, our study revealed that there are demographic and economic differences regarding the adherence to pharmacological treatment. The predominance of this type of treatment was similar among men and women, but different between the macro-regions of the country, suggesting a barrier in access: the prevalence of pharmacological treatment was higher in the Southeast and lower in the North, among patients with chronic bronchitis; and higher in the South and lower in the Northeast, among emphysema patients. A probable explanation for this difference is that the program *“Aqui Tem Farmácia Popular*” (We have Popular Pharmacy) – considered one of the branches of the Popular Pharmacy Program of Brazil, in 2012 – was predominant, in terms of coverage and expansion of its units, in the Southeast region with 49.5% of the accounted pharmacies, whereas the Northeast had only 11%^20^.

Regarding pharmacological treatment, especially among individuals who reported only emphysema, we found a statistically significant difference regarding race/skin color and wealth index. However, since the number of Asians (n = 5) and Indigenous (n = 7) individuals was small, our results are limited. Drummond et al.^21^observed that non-white individuals are 43% more likely to not obtain medications when compared to white individuals.

Although the Family Health Strategy (*Estratégia Saúde da* Família – ESF) contributes to the improvement of diagnosis and monitoring of chronic conditions, the challenges still persist^22^. Problems such as the difficulty of understanding and the adherence of families to the guidelines limit the efficiency of the ESF, and it could explain the low percentages of pharmacological treatment found in individuals with chronic bronchitis, registered in FHU^23^. The low adherence, although not measured in our study, is also an important factor regarding the control of symptoms of the disease. One study^24^ investigated the use of metered-dose inhaler in Latin America and concluded that low adherence to treatment was associated with increased frequency of exacerbations, as well as the negative impact of COPD on the individual’s life. These patients, in turn, had low educational level. In our study, a lower prevalence of medication use was found at the lowest educational levels, except for the emphysema subgroup, in which this treatment was more frequent among the illiterate.

Regarding the wealth index, the underutilization of medicines remains higher among the poorest. Although the program “*Aqui Tem Farmácia Popular*” has contributed positively by increasing access to medicines, not everyone is included in the list for gratuity, meaning that many individuals still have to pay to acquire the medication^18^.

Physical therapy, which improves pulmonary function, decreases dyspnea, and increases exercise and physical activity capacity^25^, was used by only 6.9% of individuals who reported having COPD. Among the disease subgroups, this type of treatment was more frequent in individuals with emphysema (8.8%) than in individuals with chronic bronchitis (4.7%). We observed that the use of physical therapy was higher among the richest.

Due to the need for medical referral for physical therapy within the public healthcare network, this treatment becomes relatively rare: approximately one in five users uses this service (19%)^26^. Despite the existence of strong evidence regarding the reduction (4.27 days) of the number of days of hospitalization, readmission, and mortality of individuals with COPD^27^, physical therapy is a reality only for a minority of the population, evidencing not only the lack of knowledge about the guidelines of Primary Health Care but also the limited access to this type of treatment^28^.

As another tool for COPD management is the oxygen therapy. According to the II *Consenso Brasileiro sobre Doença Pulmonar Obstrutiva Crônica*^29^, oxygen therapy is recommended in the most severe phase of the disease, which is a reality for the minority of the population (1.33%)^9^, and this is another possible explanation for the low prevalence of this type of treatment, which was more frequent among those who self-reported their health as “very bad.”

Notably, there were no differences between access to treatment and having insurance or not, except in relation to the use of oxygen therapy, which was 10 times more frequent among those who did not have health insurance. This type of procedure was also seven times more frequent in the illiterate population. These results highlight the importance of the SUS in promoting equity and supporting the population that needs it most^30^. The use of oxygen consists of a very expensive item that encumbers families. Thus, the SUS provides this resource for home use and in health units, in addition to other items for pharmacological treatment, as well as physical therapy^30^.

Some limitations of this study must be emphasized. Since this is a cross-sectional study, it is not known whether exposure preceded the outcome and, thus, some statements may be subject to reverse causality, such as the relationship between smoking and treatment. Moreover, as the information was collected in a self-reported manner, there may be information bias, minimizing some differences found. The severity of COPD was not an information available in the PNS. Self-assessment of health, however, can be considered a proxy. Diagnosis depends on access to health services and this information was not analyzed; the access measured in the PNS was related to any diseases, not just COPD.

As a strong point of the study, we highlight the fact that the sample is representative of the Brazilian population, allowing us to obtain extremely important results for the evaluation of health conditions in a national overview, assisting administrators in the formulation of health policies and actions to cope with COPD.

Our findings indicate that the prevalence of tools for the management of chronic obstructive pulmonary disease are still below ideal, contributing to high economic and social costs, direct or indirect. This study highlights the alarming situation, emphasizing the need for strategic actions for greater and better indication and adherence to the proposed treatments.
