# Supplementary material for: Treatments used by chronic obstructive pulmonary disease patients in Brazil: National Survey of Health, 2013
Source: Rev Saude Publica. 2022 Nov 18;56:119. doi: 10.11606/s1518-8787.2022056004090 (PMC9749731; doi:10.11606/s1518-8787.2022056004090)
Supplement: Translation

# Translation: Tratamentos utilizados por portadores de DPOC no Brasil: Pesquisa Nacional de Saúde, 2013

**Authors:** Gabriela Ávila Marques, Paula Duarte de Oliveira, Marina Montzel, Ana Maria Baptista Menezes, Deborah Carvalho Malta, Luciana Monteiro Vasconcelos Sardinha, Fernando César Wehrmeister

**Affiliations:** I Brasil Universidade Federal de Pelotas. Faculdade de Medicina. Programa de Pós-Graduação em Epidemiologia. Pelotas, RS, Brasil; II Brasil Universidade Federal de Pelotas. Faculdade de Medicina. Departamento de Medicina Social. Pelotas, RS, Brasil; III Brasil Universidade Federal de Minas Gerais. Escola de Enfermagem. Departamento de Enfermagem Materno-Infantil e Saúde Pública. Belo Horizonte, MG, Brasil; IV Brasil Ministério da Saúde. Brasília, DF, Brasil

**Keywords:** Doença Pulmonar Obstrutiva Crônica, epidemiologia, Bronquite Crônica, Enfisema Pulmonar, Gerenciamento Clínico, Inquéritos Epidemiológicos

## Abstract

**OBJETIVO:**

Estimar a prevalência dos tratamentos utilizados para o manejo da doença pulmonar obstrutiva crônica (DPOC) na população adulta brasileira.

**MÉTODOS:**

Estudo transversal de base populacional com dados oriundos da Pesquisa Nacional de Saúde de 2013, incluindo indivíduos com 40 anos ou mais, com diagnóstico médico autorreferido de DPOC, bronquite crônica e/ou enfisema, os quais foram questionados sobre tratamentos utilizados para o manejo da doença.

**RESULTADOS:**

Foram entrevistados 60.202 adultos, dos quais 636 tinham 40 ou mais anos de idade e haviam referido diagnóstico médico de DPOC, enfisema ou bronquite crônica. Menos da metade (49,4%) da população diagnosticada relatou utilizar algum tipo de tratamento, havendo diferenças quanto à macrorregião do país (Sul 53,8% – Nordeste 41,2%, p = 0,007). O tratamento medicamentoso foi o mais referido e portadores de enfisema apresentaram a maior proporção de mais de um tipo de tratamento utilizado. Entre os indivíduos que declararam ter apenas bronquite crônica, 55,1% (IC95% 48,7–61,4) usavam medicamento, 4,7% (IC95% 2,6–8,3) realizavam fisioterapia e 6,0% (IC95% 3,6–9,9) oxigenoterapia. Por outro lado, entre os enfisematosos, 44,1% (IC95% 36,8–51,7) realizavam tratamento medicamentoso, 8,8% (IC95% 5,4–14,2) fisioterapia e 10,0% (IC95% 6,3–15,6) oxigenoterapia.

**CONCLUSÕES:**

As prevalências de tratamentos para o manejo da DPOC estavam aquém do ideal em 2013. O medicamentoso foi o principal tipo de tratamento, seguido de oxigenoterapia e fisioterapia.

## INTRODUÇÃO

As doenças crônicas não transmissíveis, incluindo as doenças respiratórias crônicas, são as principais causas de morbidade e mortalidade no Brasil. A doença pulmonar obstrutiva crônica (DPOC) é a terceira causa de morte no mundo, sendo responsável por mais de três milhões de óbitos por ano^1,2^.

Abrangendo a bronquite crônica e o enfisema pulmonar, a DPOC é caracterizada pela obstrução crônica das vias aéreas inferiores, ocasionando sintomas, como sibilância, dispneia e tosse produtiva^2^. A minimização da exposição aos fatores de risco, articulada à educação em saúde do paciente e à adesão ao tratamento recomendado, possui um importante papel para reduzir a frequência e a severidade das crises/exacerbações^2^.

O protocolo de tratamento de DPOC, no Brasil, é seguido por toda a rede do Sistema Único de Saúde (SUS). Dentre os diferentes tipos de tratamento, o farmacológico é considerado um dos principais pilares no manejo da doença^3^. A fisioterapia respiratória, a oxigenoterapia e/ou uso da ventilação não invasiva, conforme gravidade e indicação, também demonstram ser estratégias com grande potencial para minimizar os sintomas respiratórios, melhorar a qualidade de vida e reduzir o risco de mortalidade^4^.

A ausência ou tratamento inadequado pode desencadear internações desnecessárias, maiores custos diretos e indiretos à população e elevada mortalidade^5^. Apenas uma pequena parcela da população realiza algum tipo de tratamento, como mostrou um inquérito^6^ sobre DPOC, o qual concluiu que, no Brasil, especialmente em São Paulo, 83,3% das pessoas com doença pulmonar obstrutiva crônica não recebiam tratamento farmacológico.

Frente a este cenário, o objetivo do presente estudo foi estimar a prevalência dos tratamentos utilizados para o manejo da DPOC de acordo com variáveis sociodemográficas, comportamentais e utilização de serviços de saúde em uma amostra representativa da população adulta do Brasil.

## MÉTODOS

### Desenho do Estudo e Fonte de Dados

Trata-se de um estudo transversal de base populacional com dados da Pesquisa Nacional de Saúde (PNS) de 2013. A PNS é realizada pelo Instituto Brasileiro de Geografia e Estatística (IBGE), em parceria com o Ministério da Saúde, e possui como objetivo a avaliação do desempenho do sistema e das condições de saúde dos brasileiros. Para garantir a representatividade da população nacional, foi utilizada amostragem por conglomerados em três estágios: setores censitários, domicílios e indivíduos adultos (18 ou mais anos de idade)^7^.

O questionário aplicado incluiu questões sobre o domicílio, as visitas domiciliares realizadas pela equipe de Saúde da Família e os agentes de saúde, relativas às características da totalidade de moradores do domicílio e questões individuais sobre saúde ao morador. Para este estudo foi utilizado o módulo intitulado “Doenças Crônicas”, no qual a população foi questionada sobre o diagnóstico médico de DPOC e os tratamentos utilizados para o controle da doença. Mais detalhes da PNS estão disponíveis em publicações prévias^8^.

Para este estudo, foram incluídos indivíduos com 40 anos ou mais (seguindo o ponto de corte de faixa etária de outros estudos sobre DPOC)^9^, que responderam positivamente à pergunta “Q116. Algum médico já lhe deu o diagnóstico de alguma doença no pulmão, tais como enfisema pulmonar? (não/sim), bronquite crônica? (não/sim) ou DPOC (doença pulmonar obstrutiva crônica)? (não/sim)”. Foi possível diferenciar a presença de bronquite crônica e enfisema, sendo criada uma variável dicotômica para cada um destes agravos.

### Tratamentos Utilizados pelos Portadores da Doença

Aos indivíduos que relataram ter DPOC, foi questionado sobre tratamentos, por meio da pergunta “Q118. O que o(a) Sr(a) faz atualmente por causa da doença no pulmão?”. Dentre as alternativas disponíveis para a resposta, estavam: “a. Usa medicamentos (inaladores, aerossol ou comprimidos); b. Usa oxigênio; c. Fisioterapia respiratória; d. Outro”, podendo o entrevistado responder a mais de uma opção. Foram criadas variáveis dicotômicas (sim/não) para cada um dos tratamentos listados e uma variável para quem realiza qualquer dos três tipos de tratamento.

### Variáveis de Exposição

Os tratamentos foram descritos de acordo com as variáveis: sexo (masculino/feminino), idade em anos (40-49, 50-59, 60 ou mais), cor da pele (branca, preta, morena/parda, amarela, indígena), tabagismo atual (não/sim), escolaridade/nível de instrução (analfabeto, fundamental incompleto, fundamental completo ou médio incompleto; médio completo ou superior incompleto, superior completo ou mais), local de moradia (urbano/rural), macrorregião do país (Norte, Nordeste, Sudeste, Sul, Centro-Oeste), índice de riqueza e variáveis relacionadas a acesso e utilização de serviços de saúde.

Foi utilizada a análise dos componentes principais para a construção do índice de riqueza^10^, independentemente do local de residência. Dentre as variáveis disponíveis, foram selecionadas 14 que faziam referência à presença ou não de um determinado bem ou facilidade, sendo elas: número de carros, aparelho de DVD, máquina de lavar, micro-ondas, telefone, computador, celular, fonte melhorada de abastecimento de água, fonte de energia elétrica, acesso à internet, número de banheiros e uso de materiais apropriados na construção do domicílio (telhado, parede, piso). Posteriormente, o índice de riqueza foi dividido em quintis, sendo o primeiro quintil (Q1) composto pelos indivíduos mais pobres e o quintil mais alto (Q5) pelos mais ricos.

As variáveis de acesso e utilização de serviços de saúde foram: cadastro do domicílio na Unidade Saúde da Família (USF) (não/sim) e posse de plano de saúde (não/sim).

### Análise Estatística

Foi realizada a descrição da amostra, por meio de frequências absolutas e relativas, segundo variáveis de exposição. Ao considerar o tipo de tratamento, estratificado pelo tipo de doença (DPOC, bronquite crônica ou enfisema), as estimativas foram expressas em frequência relativa, com intervalos de 95% de confiança (IC95%), considerando o desenho amostral da pesquisa, por meio do comando *svy*. A comparação das prevalências de cada tratamento de acordo com as variáveis de exposição foi realizada por meio do teste do χ^2^, considerando estatisticamente significativos os resultados com valor p < 0,05.

Para visualizar o uso de mais de um tipo de tratamento, concomitantemente, foram construídos Diagramas de Venn para cada subgrupo de doença. Nele, cada uma das circunferências representa um dos tratamentos (farmacológico, oxigenoterapia ou fisioterapia). Dessa maneira, é possível visualizar a proporção de cada intersecção.

Todos os dados foram analisados por meio do programa estatístico *Stata*, na versão 15.0 (StataCorp LP, College Station, TX, Estados Unidos).

### Aspectos Éticos

A PNS foi aprovada pela Comissão Nacional de Ética em Pesquisa. A participação dos indivíduos foi voluntária e a confidencialidade das informações garantida. Os participantes que concordaram em participar assinaram o Termo de Consentimento Livre e Esclarecido. Os dados estão publicamente disponíveis no site do Instituto Brasileiro de Geografia Estatística (IBGE)^8^.

## RESULTADOS

Dentre os 60.202 adultos entrevistados, 31.612 (35,2%) tinham 40 ou mais anos de idade e, desses, 636 (2,0%) relataram diagnóstico médico de DPOC. A prevalência populacional de bronquite crônica, em indivíduos com 40 anos ou mais foi de 0,9% e de enfisema, foi de 0,7%, isolados. A descrição da amostra, conforme o diagnóstico autorreferido, bem como a realização de algum tratamento, pode ser observada na Tabela 1. Entre todos os tipos de agravos avaliados, a maioria dos indivíduos tinha 60 anos ou mais, era de cor da pele branca, não fumava, possuía escolaridade baixa, era residente na zona urbana, com domicílio cadastrado na USF, sem plano de saúde e considerava sua saúde de regular a muito ruim. Quanto ao sexo, portadores de DPOC e de bronquite crônica eram na maioria mulheres, enquanto, entre enfisematosos, a maior proporção era do sexo masculino (Tabela 1). Em relação ao uso de qualquer tratamento, 47% relatam algum tratamento para DPOC, 56% para bronquite crônica e 48% para enfisema. Em portadores de DPOC, houve diferenças quanto à macrorregião do país (Sul 53,8%, Nordeste 41,2%) e quanto à autoavaliação de saúde (ruim 67,1% e muito boa 23,1%). Entre portadores de bronquite crônica, padrão semelhante foi encontrado para macrorregião do país e maior proporção de qualquer tratamento entre aqueles com domicílio não cadastrado na USF (66,3%). Em enfisematosos, homens faziam mais tratamento do que mulheres (53,1% contra 45,9%).

**Tabela 1:** Descrição da amostra de brasileiros com diagnóstico médico autorreferido de doença pulmonar obstrutiva crônica (DPOC), bronquite crônica ou enfisema, e que referiram realizar algum tipo de tratamento, segundo variáveis sociodemográficas, comportamentais e utilização de serviços de saúde. PNS, 2013.

Variáveis	DPOC (n = 636)			Bronquite crônica (n = 234)			Enfisema (n = 170)		
		
	n	%	Realiza algum tratamento (%)	n	%	Realiza algum tratamento (%)	n	%	Realiza algum tratamento (%)
Sexo			p = 0,502			p = 0,967			p = 0,017
Masculino	269	42,3	47,2	78	33,3	48,7	96	56,5	53,1
Feminino	367	57,7	51,0	156	66,7	62,8	74	43,5	45,9
Idade, anos			p = 0,458			p = 0,905			p=0,625
40–49	133	20,9	46,6	59	25,2	55,9	16	9,4	37,5
50–59	151	23,7	49,0	63	26,9	60,3	35	20,6	42,9
≥ 60	352	55,4	50,6	112	47,9	58,0	119	70,0	53,8
Cor da pele			p = 0,105			p = 0,422			p = 0,203
Branca	332	52,2	51,8	120	51,3	61,7	92	54,1	53,3
Preta	50	7,9	42,0	21	9,0	52,4	5	2,9	40,0
Amarela	5	0,8	40,0	1	0,4	100,0	1	0,6	0,0
Morena/parda	242	38,1	47,5	91	38,9	54,9	71	41,8	47,9
Indígena	7	1,1	57,1	1	0,4	0,0	1	0,6	0,0
Tabagismo atual			p = 0,104			p = 0,357			p = 0,323
Não	482	75,8	52,3	172	73,5	64,5	127	74,7	55,1
Sim	154	24,2	40,3	62	26,5	40,3	43	25,3	34,9
Escolaridade			p = 0,081			p = 0,113			p = 0,055
Analfabeto	144	22,6	52,1	54	23,1	55,6	43	25,3	58,1
Fundamental incompleto	245	38,5	45,7	91	38,9	48,4	66	38,8	43,9
Fundamental completo ou médio incompleto	70	11,0	48,6	22	9,4	72,7	20	11,8	55,0
Médio completo ou superior incompleto	102	16,0	52,9	36	15,4	75,0	27	15,9	55,6
Superior completo	75	11,8	52,0	31	13,3	61,3	14	8,2	35,7
Local de moradia			p = 0,300			p = 0,639			p = 0,195
Urbano	550	86,5	49,8	198	84,6	59,1	150	88,2	50,0
Rural	86	13,5	46,5	36	15,4	52,8	20	11,8	50,0
Macrorregião			p = 0,007			p < 0,001			p = 0,094
Norte	80	12,6	50,0	29	12,4	51,7	20	11,8	60,0
Nordeste	131	20,6	41,2	33	14,1	45,5	39	22,9	38,5
Sudeste	193	30,4	51,8	75	32,1	70,1	44	25,9	52,3
Sul	143	22,5	53,8	62	26,5	56,5	41	24,1	53,7
Centro-Oeste	89	14,0	48,3	35	15,0	51,4	26	15,3	50,0
Quintil de riquezas			p = 0,185			p = 0,429			p = 0,071
Q1 (mais pobre)	94	14,8	40,4	33	14,1	42,4	24	14,1	37,5
Q2	159	25,0	49,7	58	24,8	51,7	49	28,8	51,0
Q3	153	24,1	48,4	62	26,5	59,7	42	24,7	50,0
Q4	102	16,0	48,0	38	16,2	68,4	18	10,6	44,4
Q5 (mais rico)	128	20,1	57,8	43	18,4	67,4	37	21,8	59,5
Domicílio cadastrado na USF			p = 0,625			p < 0,001			p = 0,254
Não	277	43,6	47,3	98	41,9	66,3	73	42,9	46,6
Sim	359	56,5	51,0	136	58,1	52,2	97	57,1	52,6
Plano de saúde			p = 0,635			p = 0, 296			p = 0,499
Não	407	64,0	50,4	153	65,4	57,5	110	64,7	51,8
Sim	229	36,0	47,6	81	34,6	59,3	60	35,3	46,7
Autoavaliação da saúde			p = 0,023			p = 0,461			p = 0,283
Muito boa	26	4,1	23,1	9	3,9	22,2	4	2,4	0
Boa	181	28,5	40,9	58	24,8	53,4	42	24,7	47,6
Regular	300	47,2	50,7	119	50,9	58,8	83	48,8	48,2
Ruim	85	13,4	67,1	33	14,1	72,7	28	16,5	64,3
Muito ruim	44	6,9	56,8	15	6,4	60,0	28	7,7	53,8

USF: unidade de saúde da família.

A Figura apresenta as proporções de cada tratamento (farmacológico, fisioterapêutico e oxigenoterapia) nos três subgrupos. Em todos, o medicamentoso foi o mais referido. Os portadores de enfisema apresentaram a maior proporção de mais de um tipo de tratamento utilizado. Entre os indivíduos avaliados, 44,8% declararam usar medicamentos para o manejo da doença, com diferenças apenas por macrorregião do país, sendo a Nordeste com a menor prevalência (27,5%; IC95% 18,3–39,1) e a Sudeste com a maior (55,2%; IC95% 44,8–65,1) (Tabela 2).

**Figura:** Diagrama de Venn com as devidas proporções dos tratamentos utilizados para o controle da doença pulmonar obstrutiva crônica (DPOC). PNS, 2013.

**Tabela 2:** Distribuição da prevalência*a de uso de medicamentos em brasileiros portadores da doença pulmonar obstrutiva crônica (DPOC), bronquite crônica ou enfisema, segundo variáveis sociodemográficas, comportamentais e utilização de serviços de saúde. PNS, 2013.

Variáveis	Medicamento					

	DPOC		Bronquite crônica		Enfisema	
		
	%	IC95%	%	IC95%	%	IC95%
Sexo		p = 0,625		p = 0,979		p = 0,039
Masculino	49,5	39,6–59,4	62,7	47,4–75,8	53,7	38,0–68,6
Feminino	46,1	37,6–54,9	62,4	49,4–73,9	29,1	15,9–47,1
Idade, anos		p = 0,357		p = 0,941		p = 0,307
40–49	47,2	33,8–61,0	64,7	41,8–82,4	40,7	14,7–73,3
50–59	39,1	28,0–51,4	60,0	41,6–75,9	24,1	11,0–44,9
≥ 60	50,8	42,0–59,6	62,4	48,4–74,7	48,0	33,6–62,8
Cor da pele		p = 0,039		p = 0,363		p = 0,015
Branca	54,5	45,9–62,8	67,9	55,4–78,2	52,3	36,5–67,6
Preta	30,6	14,5–53,4	57,4	27,6–82,6	6,2	0,6–41,4
Amarela	66,4	13,2–96,2	100,0	–	0,0	–
Morena/parda	38,0	28,0–49,0	53,3	35,3–70,4	30,8	18,1–47,4
Indígena	45,4	7,1–90,0	0,0	–	0,0	–
Tabagismo atual		p = 0,134		p = 0,366		p = 0,413
Não	50,6	42,9–58,2	65,3	53,0–75,7	46,9	33,2–61,2
Sim	38,4	26,3–52,2	54,8	35,3–72,8	34,8	15,4–61,0
Escolaridade		p = 0,106		p = 0,125		p = 0,168
Analfabeto	51,6	38,0–65,0	50,1	29,3–70,8	66,1	42,4–83,7
Fundamental incompleto	42,8	32,8–53,4	54,2	38,9–68,8	36,8	21,5–55,3
Fundamental completo ou médio incompleto	37,2	20,3–57,9	68,9	33,2–90,8	30,3	9,4–64,6
Médio completo ou superior incompleto	47,2	31,9–63,0	73,3	49,5–88,5	39,1	16,3–67,9
Superior completo	69,4	52,0–82,6	82,3	63,0–92,7	43,6	16,1–75,7
Local de moradia		p = 0,254		p = 0,69		p = 0,009
Urbano	48,4	41,5–55,4	62,9	52,1–72,6	45,4	33,1–58,3
Rural	38,6	25,0–54,2	58,2	36,8–76,9	14,9	5,2–36,0
Macrorregião		p = 0,005		p < 0,001		p = 0,087
Norte	33,0	19,7–49,7	27,3	12,0–50,9	60,0	31,4–83,1
Nordeste	27,5	18,3–39,1	32,6	14,8–57,4	17,4	7,2–36,3
Sudeste	55,2	44,8–65,1	79,8	67,5–88,3	42,8	23,8–64,1
Sul	49,3	37,2–61,4	49,8	32,3–67,3	57,8	36,4–76,7
Centro-Oeste	44,0	31,2–57,6	55,5	35,5–73,9	44,7	23,1–68,5
Quintil de riquezas		p = 0,138		p = 0,444		p = 0,044
Q1 (mais pobre)	32,9	20,7–48,1	42,4	20,8–67,3	21,3	6,7–50,5
Q2	42,2	29,8–55,6	59,9	39,9–77,1	39,6	20,0–63,1
Q3	47,5	34,7–60,6	56,9	36,7–74,9	44,8	21,8–70,3
Q4	44,0	28,6–60,7	65,6	41,3–83,8	24,3	7,3–56,7
Q5 (mais rico)	61,1	48,5–72,4	74,1	52,9–87,9	76,9	57,9–88,9
Domicílio cadastrado na USF		p = 0,473		p < 0,001		p = 0,455
Não	50,3	40,8–59,7	79,3	67,7–87,5	38,4	22,3–57,5
Sim	45,7	37,2–54,4	49,5	36,9–62,2	47,7	32,3–63,6
Plano de saúde		p = 0,505		p = 0,278		p = 0,738
Não	49,3	40,9–57,7	66,9	53,9–77,7	42,5	28,7–57,7
Sim	44,7	34,8–55,1	55,8	39,7–70,7	47,0	26,9–68,2
Autoavaliação da saúde		p = 0,051		p = 0,493		p = 0,299
Muito boa	62,6	29,9–86,8	77,9	32,6–96,2	0,0	–
Boa	34,8	24,4–47,0	58,6	38,6–76,2	32,4	13,9–58,7
Regular	48,2	39,1–57,5	57,4	42,9–70,8	45,0	29,2–61,8
Ruim	58,4	41,9–73,3	77,5	55,9–90,3	42,5	18,6–70,4
Muito ruim	67,4	45,1–83,8	71,6	37,2–91,5	73,9	35,5–93,6

USF: unidade de saúde da família.

^a^ Ponderada pelo peso do indivíduo selecionado para a amostra.

Valores com significância estatística estão apresentados em negrito.

O tratamento fisioterapêutico (Tabela 3) era realizado por 6,9% (IC95% 5,2–9,2), 4,7% (IC95% 2,6–8,3) e 8,8% (IC95% 5,4–14,2) dos portadores de DPOC, de bronquite crônica e entre os enfisematosos, respectivamente. Apenas 1,3% (IC95% 0,5–3,0) dos tabagistas atuais faziam fisioterapia comparado a 6,8% dos não tabagistas (p < 0,001). Entre enfisematosos, diferenças por quintil de riqueza foram observadas, sendo que o quintil mais rico teve 23,7% (IC95% 9,3–48,7), enquanto nos outros quintis essa prevalência foi inferior a 6% (p = 0,003) (Tabela 3).

**Tabela 3:** Distribuição da prevalênciaa de realização de fisioterapia em brasileiros portadores da doença pulmonar obstrutiva crônica (DPOC), bronquite crônica ou enfisema, segundo variáveis sociodemográficas, comportamentais e utilização de serviços de saúde. PNS, 2013.

Variáveis	Fisioterapia					

	DPOC		Bronquite crônica		Enfisema	
		
	%	IC95%	%	IC95%	%	IC95%
Sexo		p = 0,328		p = 0,569		p = 0,884
Masculino	6,9	3,6–12,8	5,7	1,1–25,8	6,3	2,5–15,4
Feminino	4,4	2,4–8,1	3,3	1,3–8,2	7,1	1,8–24,1
Idade (anos)		p = 0,886		p = 0,012		p = 0,09
40–49	4,9	1,6–13,7	2,0	0,6–6,7	14,2	2,0–57,7
50–59	4,7	1,6–12,9	0,1	0,0–1,0	18,9	5,3–49,4
≥ 60	6,0	3,4–10,4	6,6	2,4–17,2	3,2	1,4–7,2
Cor da pele		p = 0,010		p = 0,244		p = 0,14
Branca	4,5	2,3–8,6	4,5	1,3–14,2	4,7	1,3–14,8
Preta	22,7	8,2–49,1	19,6	2,9–66,2	31,2	3,6–84,5
Amarela	0,0	–	0,0	–	0,0	–
Morena/Parda	4,2	2,3–7,7	1,8	0,6–6,0	7,4	2,7–18,7
Indígena	0,0	–	0,0	–	0,0	–
Tabagismo atual		p < 0,001		p = 0,025		p = 0,092
Não	6,8	4,3–10,8	5,3	2,1–12,9	8,0	3,4–17,7
Sim	1,3	0,5–3,0	0,7	0,1–4,7	2,4	0,7–8,1
Escolaridade		p = 0,690		p = 0,476		p = 0,841
Analfabeto	4,7	1,6–13,1	2,8	0,4–18,0	7,1	1,3–31,5
Fundamental incompleto	6,3	3,1–12,6	6,0	1,4–21,7	5,8	1,5–19,4
Fundamental completo ou médio incompleto	2,4	0,7–7,6	11,4	0,1–8,8	4,3	0,7–22,7
Médio completo ou superior incompleto	4,4	1,7–10,7	0,2	0,0–1,5	7,6	1,8–26,6
Superior completo	7,8	3,1–18,2	6,1	1,4–22,5	17,0	36,3–52,5
Local de moradia		p = 0,818		p = 0,238		p = 0,525
Urbano	5,4	3,4–8,7	3,5	1,2–9,8	7,0	3,1–14,6
Rural	6,3	1,9–19,2	10,0	2,1–36,1	0,0	–
Macrorregião		p = 0,508		p = 0,246		p = 0,957
Norte	10,5	4,1–24,4	14,7	4,0–41,9	9,4	1,8–37,8
Nordeste	3,3	1,2–8,6	0,0	–	5,8	13,0–22,1
Sudeste	4,6	2,2–9,4	2,5	0,7–8,9	5,7	1,5–19,6
Sul	7,6	3,2–17,0	8,5	2,2–27,7	7,2	1,4–29,5
Centro-Oeste	6,6	2,1–18,5	0,0	–	10,2	1,9–39,3
Quintil de riquezas		p = 0,480		p = 0,222		p = 0,003
Q1 (mais pobre)	1,8	3,8–8,0	0,0	–	1,5	0,2–11,2
Q2	4,0	1,7–8,8	4,1	0,8–18,0	0,2	0,0–1,2
Q3	4,4	1,6–11,2	4,4	1,2–14,8	5,5	0,8–29,3
Q4	8,5	3,1–21,1	11,7	2,3–43,5	3,1	0,8–11,2
Q5 (mais rico)	7,0	3,1–15,2	0,5	0,1–3,7	23,7	9,3–48,7
Domicílio cadastrado na USF		p = 0,795		p = 0,762		p = 0,301
Não	5,1	2,6–9,8	4,7	1,7–12,6	4,0	1,5–10,8
Sim	5,8	3,1–10,4	3,6	0,8–14,7	8,3	3,1–20,5
Plano de saúde		p = 0,528		p = 0,672		p = 0,322
Não	4,9	2,5–9,4	4,6	1,4–14,1	4,8	1,4–14,9
Sim	6,5	3,7–11,3	3,2	0,9–10,9	10,3	3,6–26,3
Autoavaliação da saúde		p = 0,772		p = 0,477		p = 0,79
Muito boa	0,0	–	0,0	–	0,0	–
Boa	4,7	1,6–12,9	9,2	2,1–31,9	4,8	1,5–14,3
Regular	5,7	3,0–10,4	2,3	0,7–7,6	7,2	2,5–19,1
Ruim	8,6	3,2–21,2	3,2	0,8–12,5	10,9	1,5–49,3
Muito ruim	5,1	1,6–15,4	4,0	0,5–26,3	2,1	0,2–15,6

USF: unidade de saúde da família.

^a^ Ponderada pelo peso do indivíduo selecionado para a amostra.

Valores com significância estatística estão apresentados em negrito.

O tratamento com oxigênio foi utilizado por 7,2% (IC95% 5,5–9,5) dos portadores de DPOC, 6,0% (IC95% 3,6–9,9) dos indivíduos com bronquite crônica e 10,0% (IC95% 6,3–15,6) dos enfisematosos. A distribuição das prevalências de oxigenoterapia pode ser observada na Tabela 4. Ainda, o uso de oxigênio foi maior entre analfabetos que referiram DPOC (14,4%).

**Tabela 4:** Distribuição da prevalênciaa de oxigenoterapia em brasileiros portadores da doença pulmonar obstrutiva crônica (DPOC), bronquite crônica ou enfisema, segundo variáveis sociodemográficas, comportamentais e utilização de serviços de saúde. PNS, 2013.

Variáveis	Oxigenoterapia					

	DPOC		Bronquite crônica		Enfisema	
		
	%	IC95%	%	IC95%	%	IC95%
Sexo		p = 0,050		p = 0,626		p < 0,001
Masculino	10,5	5,4–19,5	8,3	2,4–25,4	17,0	7,2–35,3
Feminino	4,1	1,9–8,4	5,6	1,9–15,4	2,2	0,8–5,6
Idade, anos		p = 0,309		p = 0,053		p = 0,896
40–49	4,2	1,3–12,2	1,3	0,3–5,8	13,6	2,3–51,5
50–59	4,6	1,6–12,7	4,1	1,1–14,1	14,7	3,2–47,5
≥ 60	8,8	4,7–15,9	9,9	3,7–23,6	10,2	3,4–26,6
Cor da pele		p = 0,480		p = 0,784		p = 0,163
Branca	8,8	4,7–15,9	7,7	2,6–20,6	13,3	5,1–30,8
Preta	8,3	1,5–34,4	1,6	0,2–11,5	31,2	3,6–84,5
Amarela	0,0	–	0,0	–	0,0	–
Morena/parda	3,5	1,8–6,8	5,1	1,9–12,9	2,7	1,1–6,3
Indígena	0,0	–	0,0	–	0,0	–
Tabagismo atual		p < 0,001		p < 0,001		p < 0,001
Não	8,9	5,3–14,6	8,5	3,7–18,4	14,5	6,4–29,7
Sim	0,7	0,3–1,9	0,5	0,1–3,6	0,8	0,1–4,0
Escolaridade		p = 0,012		p = 0,275		p = 0,332
Analfabeto	14,4	6,4–29,2	11,7	2,7–38,8	21,3	6,4–51,9
Fundamental incompleto	7,7	3,6–15,4	9,3	3,4–23,2	10,9	3,3–30,3
Fundamental completo ou médio incompleto	0,7	0,1–3,3	2,0	0,4–10,5	0,0	–
Médio completo ou superior incompleto	1,7	0,5–5,1	0,0	–	24,5	0,5–11,0
Superior completo	2,1	0,6–6,7	1,4	0,2–9,9	0,0	–
Local de moradia		p = 0,546		p = 0,772		p = 0,625
Urbano	7,1	4,1–11,9	6,6	2,7–14,9	11,0	4,6–24,1
Rural	5,3	2,4–11,3	5,2	1,3–18,9	15,3	5,1–38,0
Macrorregião		p = 0,280		p = 0,756		p = 0,075
Norte	6,4	2,1–17,8	8,0	1,2–39,2	13,6	3,1–43,4
Nordeste	2,5	1,1–5,7	2,1	0,4–11,3	1,7	0,4–6,9
Sudeste	9,2	4,5–18,0	8,0	2,7–21,1	19,7	6,9–44,7
Sul	5,3	1,8–14,9	5,3	0,7–29,7	7,4	1,8–25,6
Centro–Oeste	8,1	3,8–16,4	6,8	1,5–25,1	1,8	0,2–12,2
Quintil de riquezas		p = 0,366		p = 0,017		p = 0,343
Q1 (mais pobre)	3,9	1,4–10,5	0,6	0,1–4,3	8,2	1,8–30,7
Q2	3,1	1,3–7,1	1,1	0,2–7,5	1,4	0,4–4,9
Q3	11	4,6–24,3	10,6	2,9–32,2	17,7	4,8–48,1
Q4	8,5	3,2–20,7	18,4	5,8–45,2	10,2	1,5–46,1
Q5 (mais rico)	6,0	1,7–19,0	0,9	0,1–6,6	18,7	3,9–56,6
Domicílio cadastrado na USF		p = 0,524		p = 0,571		p = 0,648
Não	5,7	2,4–12,6	5,0	2,0–12,0	8,5	1,5–35,6
Sim	7,9	14,5–	7,5	2,4–21,3	13,0	5,1–29,1
Plano de saúde		p = 0,646		p = 0,002		p = 0,558
Não	7,6	4,1–13,6	9,8	4,1–21,7	9,3	3,2–24,5
Sim	5,9	2,3–14,1	1,2	0,3–4,9	14,9	4,2–41,5
Autoavaliação da saúde		p = 0,295		p = 0,040		p = 0,085
Muito boa	0,0	–	0,0	–	0,0	–
Boa	4,1	1,3–12,3	7,0	1,2–31,1	1,3	0,3–6,7
Regular	6,4	2,9–13,6	4,5	1,9–10,4	12,5	3,8–33,7
Ruim	12,3	4,9–27,6	1,8	0,2–12,7	28,1	9,3–59,8
Muito ruim	18,0	4,6–50,4	39,6	8,8–81,6	3,0	0,6–13,9

USF: unidade de saúde da família.

^a^ Ponderada pelo peso do indivíduo selecionado para a amostra.

Valores com significância estatística estão apresentados em negrito.

## DISCUSSÃO

A proporção de brasileiros portadores de doença pulmonar obstrutiva crônica que declararam realizar/usar algum tratamento estava aquém do ideal em 2013, nos três subgrupos analisados (DPOC, bronquite crônica e enfisema). Foi encontrada a predominânciade mulheres entre os portadores de DPOC e bronquite crônica, divergente de outros estudos, possivelmente como resultado de interpretações errôneas e relacionadas a outras doenças respiratórias, sejam elas crônicas (como no caso da asma), ou agudas (bronquite aguda). Historicamente, mulheres utilizam mais serviços de saúde quando comparadas com os homens, com maiores chances de obter o diagnóstico^11^.

O tratamento medicamentoso foi o mais prevalente (44,7%), seguido de oxigenoterapia (7,2%) e fisioterapia (6,9%). Ainda, dentre os que utilizavam pelo menos um dos tipos de tratamento questionados, foram observadas importantes diferenças por sexo, cor da pele, história de tabagismo, escolaridade, macrorregião do país, bem como índice de riqueza e autoavaliação da saúde dos indivíduos. De uma maneira geral, enquanto a região Sudeste apresentou as maiores prevalências dos diferentes tipos de tratamento, a região Nordeste apresentou as menores/piores.

As doenças crônicas não trasmissíveis correspondem a 72% das causas de morte no Brasil, das quais, 5,8% ocorrem devido às doenças respiratórias crônicas^12^. Muitas dessas mortes poderiam ser evitadas com maior atenção e investimento na tríade diagnóstico-indicação-adesão ao esquema terapêutico. A menor adesão ao tratamento recomendado, especialmente ao farmacológico, está associada ao acréscimo em até 58% no risco de hospitalização e em até 40% no de morte^13^. Adicionalmente, o uso contínuo dos fármacos está associado a uma sobrevida de até cinco anos em pacientes com DPOC moderada ou grave^14^.

Embora muitos avanços tenham ocorrido nos últimos anos, a cessação do tabagismo ainda é o principal fator para tratar e controlar a doença^15,16^. No presente estudo, apesar das altas prevalências de tabagismo, fumantes foram os que menos fizeram algum tipo de tratamento, com diferenças bem marcadas.

A bronquite crônica e o enfisema geralmente ocorrem de forma simultânea, porém, possuem definições distintas e um mesmo indivíduo pode apresentar diferentes graus de comprometimento^17^. Nesta análise, apenas uma pequena parcela da população realizava terapias combinadas (Figura), especialmente a que resulta da articulação entre tratamento medicamentoso e fisioterapêutico, a qual, diferente da oxigenoterapia, é mais frequente em estágios menos avançados da doença^3^.

Um estudo realizado com dados da Pesquisa Nacional sobre Acesso, Utilização e Promoção do Uso Racional de Medicamentos no Brasil^18^ observou que, dentre os indivíduos que possuíam diagnóstico de doenças respiratórias crônicas, apenas 41,9% referiram ter indicação de tratamento farmacológico. Nestes, apenas 77,1% utilizavam os medicamentos, sugerindo uma importante lacuna entre indicação e adesão ao tratamento, influenciando no controle da morbidade e mortalidade da doença^18,19^. No mesmo sentido, o presente estudo revelou que há diferenças demográficas e econômicas quanto à adoção do tratamento medicamentoso. A predominância desse tipo de tratamento foi similar entre homens e mulheres, mas diferente nas macrorregiões do país, sugerindo uma barreira no acesso: a prevalência do tratamento farmacológico foi maior na região Sudeste e menor na Norte, em portadores de bronquite crônica; maior na Sul e menor na Nordeste, nos portadores de enfisema. Uma provável explicação para essa diferença é que o chamado “*Aqui Tem Farmácia Popular*”, considerado uma das vertentes do Programa Farmácia Popular do Brasil, no ano de 2012 era, em relação à cobertura e expansão de suas unidades, predominante na região Sudeste (49,5%) e de apenas 11% na região Nordeste^20^.

Ainda em relação ao tratamento farmacológico, em especial os indivíduos que relataram apenas enfisema, foi encontrada uma diferença estatisticamente significativa no que tange à cor da pele e ao índice de riquezas. Como o número de indivíduos de cor da pele amarela (n = 5) e indígena (n = 7) era pequeno, não há precisão suficiente nesse estudo. Drummond e colaboradores^21^observaram que indivíduos de cor da pele não branca possuem 43% mais chances de não obter medicamentos, quando comparados aos brancos.

Embora a Estratégia Saúde da Família (ESF) contribua para o aprimoramento do diagnóstico e do acompanhamento das condições crônicas, desafios ainda persistem^22^. Problemas como a dificuldade de entendimento e adesão das famílias às orientações limitam a eficiência da ESF e poderiam explicar os baixos percentuais de tratamento medicamentoso encontrados em indivíduos com bronquite crônica, cadastrados em USF^23^. A baixa adesão, apesar de não ter sido mensurada no presente estudo, também é um importante fator no que tange ao controle dos sintomas da doença. Um estudo^24^, investigou a adesão ao uso de inaladores dosimetrados na América Latina e concluiu que a baixa aderência ao tratamento estava associada ao aumento na frequência das exacerbações, bem como do impacto negativo da DPOC sobre a vida do indivíduo. Estes pacientes, por sua vez, apresentavam baixa escolaridade. No presente estudo, menores prevalências de uso de medicamentos foram encontradas nos mais baixos níveis de escolaridade, com exceção do subgrupo enfisema, no qual esse tratamento foi mais frequente entre os analfabetos.

Em relação ao índice de riqueza, a subutilização de medicamentos continua maior entre os mais pobres. Embora o programa *“Aqui Tem Farmácia Popular”* tenha contribuído positivamente, aumentando o acesso a medicamentos, nem todos estão contemplados na lista dos gratuitos, fazendo com que muitos indivíduos ainda tenham que pagar para adquiri-los^18^.

A fisioterapia, que promove a melhora da função pulmonar, diminuição da dispneia, além do aumento da capacidade ao exercício e atividade física^25^, foi utilizada por apenas 6,9% dos indivíduos que relataram ter DPOC. Dentre os subgrupos das doenças, esse tipo de tratamento foi mais frequente em indivíduos com enfisema (8,8%), do que em indivíduos com bronquite crônica (4,7%). Foi observado que o uso da fisioterapiafoi maior entre os mais ricos.

Devido à necessidade de encaminhamento médico na rede pública para a realização de fisioterapia, esse tratamento torna-se, relativamente, raro: aproximadamente um em cada cinco usuários utiliza esse serviço (19%)^26^. Mesmo com a existência de fortes evidências no que tange à redução (4,27 dias) do número de dias de internação hospitalar, readmissão e mortalidade dos indivíduos portadores de DPOC^27^, esta é uma realidade para uma minoria da população, evidenciando a falta de conhecimento acerca das diretrizes da Atenção Primária á Saúde, mas também o acesso limitado a esse tipo de tratamento^28^.

Como outra ferramenta do manejo da DPOC, existe a oxigenoterapia. Segundo o II Consenso Brasileiro sobre DPOC^29^, está indicada na fase mais grave da doença, a qual é realidade para a minoria da população (1,33%)^9^, sendo esta outra possível explicação para a baixa prevalência desse tipo de tratamento, o qual foi mais frequente entre aqueles que autodeclaram sua saúde como “muito ruim”.

Destaca-se que não houve diferenças entre acesso a tratamento e ter plano ou não, exceto, em relação ao uso de oxigenoterapia, o qual foi 10 vezes mais frequente entre quem não possuía plano de saúde. Esse tipo de procedimento também foi sete vezes mais frequente na população analfabeta. Estes resultados apontam a importância do SUS em promover equidade e dar suporte à população que mais precisa^30^. O uso de oxigênio consiste em um item muito caro que onera as famílias. Assim, o SUS provê este recurso para uso domiciliar e nas unidades de saúde, além dos demais itens para tratamento medicamentoso, bem como fisioterapia^30^.

Algumas limitações do estudo devem ser ressaltadas. Como trata-se de um estudo transversal, não se sabe se a exposição precedeu o desfecho e, assim, algumas afirmações podem estar sujeitas à causalidade reversa, como a relação entre tabagismo e tratamento. Além disso, como a informação foi coletada de maneira autorreferida, pode haver viés de informação, minimizando algumas diferenças encontradas. A gravidade da DPOC não era uma informação disponível na PNS. Entretanto, a autoavaliação de saúde pode ser considerada um proxy. Sabe-se que o diagnóstico depende do acesso aos serviços de saúde e essa informação não foi analisada, pois o acesso medido na PNS era relacionado a quaisquer doenças, não apenas DPOC.

Como ponto forte do estudo, ressalta-se o fato da amostra ser representativa da população brasileira, permitindo obter resultados extremamente importantes para a avaliação das condições de saúde, no panorama nacional, auxiliando gestores na formulação de políticas e ações de saúde para o enfrentamento da DPOC.

Os achados da pesquisa indicam que as prevalências de ferramentas para o manejo da doença pulmonar obstrutiva crônica ainda estão aquém do ideal, contribuindo para altos custos econômicos e sociais, diretos ou indiretos. Esse estudo ressalta a preocupante situação, enfatizando a necessidade de ações estratégicas para a maior e melhor indicação e adesão aos tratamentos propostos.
